# Divergent serum metabolomic, skeletal muscle signaling, transcriptomic, and performance adaptations to fasted versus whey protein-fed sprint interval training

**DOI:** 10.1152/ajpendo.00265.2021

**Published:** 2021-11-08

**Authors:** Tom P. Aird, Andrew J. Farquharson, Kate M. Bermingham, Aifric O’Sulllivan, Janice E. Drew, Brian P. Carson

**Affiliations:** ^1^Physical Education and Sports Sciences, University of Limerick, Limerick, Ireland; ^2^Physical Activity for Health, Health Research Institute, University of Limerick, Limerick, Ireland; ^3^The Rowett Institute, University of Aberdeen, Aberdeen, United Kingdom; ^4^School of Agriculture and Food Science, University College Dublin, Dublin, Ireland

**Keywords:** exercise performance, mitochondrial biogenesis, skeletal muscle metabolism, whey protein

## Abstract

Sprint interval training (SIT) is a time-efficient alternative to endurance exercise, conferring beneficial skeletal muscle metabolic adaptations. Current literature has investigated the nutritional regulation of acute and chronic exercise-induced metabolic adaptations in muscle following endurance exercise, principally comparing the impact of training in fasted and carbohydrate-fed (CHO) conditions. Alternative strategies such as exercising in low CHO, protein-fed conditions remain poorly characterized, specifically pertaining to adaptations associated with SIT. Thus, this study aimed to compare the metabolic and performance adaptations to acute and short-term SIT in the fasted state with preexercise hydrolyzed (WPH) or concentrated (WPC) whey protein supplementation. In healthy males, preexercise protein ingestion did not alter exercise-induced increases in *PGC-1α*, *PDK4*, *SIRT1*, and *PPAR-δ* mRNA expression following acute SIT. However, supplementation of WPH beneficially altered acute exercise-induced *CD36* mRNA expression. Preexercise protein ingestion attenuated acute exercise-induced increases in muscle pan-acetylation and PARP1 protein content compared with fasted SIT. Acute serum metabolomic differences confirmed greater preexercise amino acid delivery in protein-fed compared with fasted conditions. Following 3 wk of SIT, training-induced increases in mitochondrial enzymatic activity and exercise performance were similar across nutritional groups. Interestingly, resting muscle acetylation status was downregulated in WPH conditions following training. Such findings suggest preexercise WPC and WPH ingestion positively influences metabolic adaptations to SIT compared with fasted training, resulting in either similar or enhanced performance adaptations. Future studies investigating nutritional modulation of metabolic adaptations to exercise are warranted to build upon these novel findings.

**NEW & NOTEWORTHY** These are the first data to show the influence of preexercise protein on serum and skeletal muscle metabolic adaptations to acute and short-term sprint interval training (SIT). Preexercise whey protein concentrate (WPC) or hydrolysate (WPH) feeding acutely affected the serum metabolome, which differentially influenced acute and chronic changes in mitochondrial gene expression, intracellular signaling (acetylation and PARylation) resulting in either similar or enhanced performance outcomes when compared with fasted training.

## Graphical abstract

**Figure:**
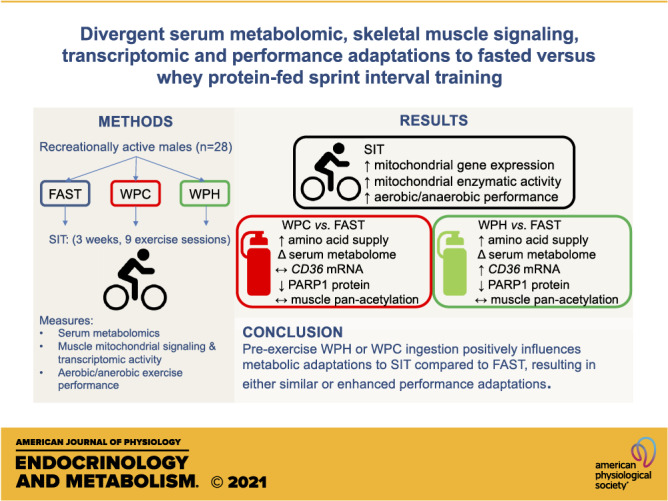

## INTRODUCTION

Skeletal muscle oxidative capacity is established as a critical determinant of maximal exercise performance (1, 2). Repeated induction of the acute signaling mechanisms that mediate muscle oxidative adaptations underpins favorable long-term physiological remodeling, including augmented exercise performance (3, 4). Nutritional strategies such as fasted-state or low carbohydrate (CHO) training, which limit exogenous substrate supply, have garnered interest as potential methods of optimizing training adaptations (5–8). Moreover, the increasing popularity of fasted training strategies in athletic populations is reflected by recent survey analyses in endurance athletes (9).

Evidence from acute exercise studies indicates that fasted endurance exercise favorably regulates muscle gene expression of *PDK4*, *UCP3*, *CPT1A*, and *CD36* compared with CHO-fed conditions (5, 8, 10). Moreover, exercise in glycogen-depleted conditions may enhance muscle mitochondrial adaptations (6, 7, 11). Changes in skeletal muscle gene expression are regulated by upstream signaling kinases that are beneficially modulated by fasting or low CHO conditions (6, 8, 12–16). The nicotinamide adenine dinucleotide (NAD^+^) biosynthetic pathway also mediates the adaptive response to exercise and low energy conditions. NAD^+^ is critical for the facilitation of electron transfer during glycolysis and oxidative phosphorylation (17) and is an essential cofactor for other metabolic processes (18). Important intermediaries of this pathway are shown to be regulated by exercise and low energy conditions (17–23). SIRT deacetylase activity is implicated in the control of mitochondrial energy metabolism, and is an example of lysine acetylation, a process which regulates energy homeostasis (24). An estimated 63% of mitochondrially localized proteins contain lysine acetylation sites (25), and although the NAD^+^-dependent nature of SIRT deacetylase activity is well characterized (26), its role in regulating global lysine acetylation is not.

Long-term studies evaluating the efficacy of fasted training for enhancing metabolic and performance adaptations are limited. Fasted endurance training promotes a shift toward increased use of fatty acids as primary fuel source during exercise, coupled with a “sparing” of muscle glycogen for high-intensity exercise (27). Augmented mitochondrial enzymatic activity and enhanced intramyocellular lipid turnover from training in fasted conditions have previously been observed (28). Curiously, little research has evaluated the acute and chronic adaptive responses under different nutritional conditions to interval-based exercise modalities, such as high-intensity interval training (HIIT) or sprint interval training (SIT). These are the two most common forms of interval training, typically classified by differences in exercise intensity (29). One study previously reported no beneficial adaptations to fasted-state HIIT in overweight/obese females (30). Though SIT induces similar physiological adaptations to endurance exercise including augmented mitochondrial biogenesis, enzymatic activity, and exercise performance (31–34), to our knowledge no previous studies have investigated the physiological adaptations to either acute or chronic SIT under such nutritional conditions.

Studies investigating fasted exercise have typically focused on comparisons with the CHO-fed state, whereas the effects of alternative feeding conditions such as a low or no CHO, protein-fed state remain poorly characterized. This is an important consideration since fasted exercise induces a net negative protein balance in the absence of amino acids, whereas net positive balance is achieved upon increasing amino acid availability (35). Preexercise protein intake provides a more favorable overall amino acid profile compared with immediate postexercise ingestion (36). Increased protein intakes are warranted when performing exercise under low CHO conditions to support rates of whole body protein synthesis, due to the greater levels of exercise-induced amino acid oxidation in low CHO conditions (37). Research studies related to protein supplementation and exercise-induced skeletal muscle adaptations have largely focused on augmenting myofibrillar adaptive responses (38), whereas evidence for the efficacy of protein supplementation to enhance mitochondrial adaptations is limited. Protein ingestion does not inhibit augmented metabolic responses associated with acute fasted exercise (39–41), and in some cases enhances fat oxidation (42). Recent in vitro evidence suggests leucine treatment stimulates or supports mitochondrial adaptations in skeletal muscle cells (43, 44).

These collective findings support a rationale that the low-CHO, but protein-fed state does not inhibit, and may potentially enhance skeletal muscle mitochondrial adaptations. However, it remains unclear to what extent preexercise protein feeding provides additional benefit compared with the overnight fasted-state, as in vivo evidence is limited. Specifically, research concerning the adaptive responses to SIT following preexercise ingestion of whey protein hydrolysates (WPH) or concentrates (WPC) is unknown. To our knowledge, no study has previously investigated the efficacy of WPH supplementation with SIT on markers of exercise-induced mitochondrial biogenesis. Recent reviews on protein hydrolysates have outlined an overall lack of evidence supporting superior anabolic properties of WPH compared with WPC, specifically with relation to studies that ensure adequate protein control groups for comparison with WPH (45). WPH ingestion augments mixed muscle protein synthesis (MPS) following resistance exercise compared with other protein sources (46), possibly due to characteristically faster rates of amino acid absorption, greater overall amino acid supply compared with nonhydrolyzed proteins (47, 48), and may increase net muscle protein balance via insulin-stimulated suppression of muscle protein breakdown (49). Coupling preliminary evidence concerning the effects of leucine and whey protein on metabolic signaling with evidence for the fasted state in augmenting skeletal muscle adaptations, a low CHO, protein-fed nutritional strategy may provide optimal conditions to maximize training adaptations. Thus, the aims of this study were twofold:

1. To determine the exercise-induced metabolic adaptations to an acute bout of SIT under fasted compared with WPC-fed and WPH-fed conditions in recreationally active males.
2. To determine the training-induced metabolic and performance adaptations to 3 wk of SIT under fasted compared with WPC-fed and WPH-fed conditions in recreationally active males.

We hypothesized that preexercise consumption of either WPH or WPC would induce divergent serum metabolomic, skeletal muscle signaling, transcriptomic, and exercise performance adaptations compared with the overnight fasted state, following both an acute bout of SIT and short-term training (3 wk).

## METHODS

### Participants

Participants were informed of all experimental procedures and potential risks before giving their written consent for participation. All experimental procedures were approved by the University of Limerick Faculty of Education and Health Sciences Research Ethics Committee (Ethics No: 2016_18_11_EHS) and were conducted in line with the World Medical Association’s Declaration of Helsinki. This study was registered at clinicaltrials.gov as NCT03570424. Twenty-eight healthy, recreationally active male participants aged 18–35 yr who were aerobically untrained (V̇o_2max_ < 50 mL·kg^−1^·min^−1^), protein sufficient [daily dietary intake ≥ 0.8 g·kg^−1^ body mass (BM)], and nonobese (fat mass index < 9 kg·m^−2^) were recruited for the study after completing preintervention medical screening (PAR-Q) and Physical Activity Readiness Questionnaires (7D-PAR).

### Baseline Screening

Prior to commencing the intervention, participants attended the laboratory after a ≥3 h fast to complete baseline screening. After measuring height and weight, body composition was characterized using bioelectrical impedance analysis (BIA) (Tanita MC-180, Amsterdam, The Netherlands). Participants subsequently completed an incremental cycle ergometer test (SRM, Jülich, Germany) until exhaustion to determine V̇o_2max_ via indirect calorimetry (Ultima CardiO_2_, MGC diagnostics, Saint Paul, MN). The protocol to determine V̇o_2max_ comprised a 5-min warm-up stage at 50 W with subsequent 30-W incremental stages every minute until exhaustion. Rate of perceived exertion (RPE) (50) and heart rate (HR) (Polar RS800 HR monitor, Kempele, Finland) were used to measure exercise intensity at the end of each stage. A true V̇o_2max_ score was achieved if two or more of the following criteria were observed: A sustained plateau or decline in V̇o_2_; HR within 85% of age predicted max (220 − participant’s age); RPE score of ≥17; Respiratory exchange ratio (RER) of greater than ≥1.10; Participant indicated volitional exhaustion.

### Experimental Design

**Figure 1.**
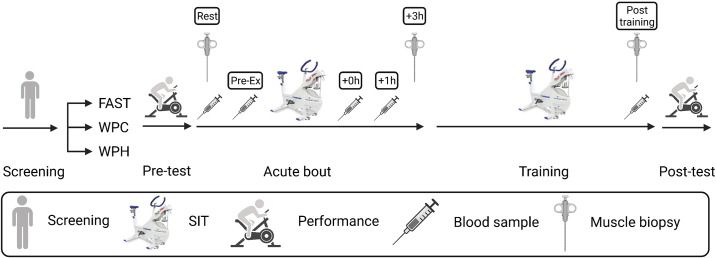
Schematic overview of experimental protocol for the study. Created with BioRender.com. FAST, fasted; Pre-Ex, prior to exercise; SIT, sprint interval training; WPC, whey protein concentrate; WPH, whey protein hydrolysate.

This study was conducted in a randomized, double-blind parallel groups design. Participants were allocated at baseline to one of the following nutritional conditions: fasted (FAST), WPC-fed (WPC), or WPH-fed (WPH). A schematic overview of the study is presented in Fig. 1.

On *day 1* of the exercise intervention, participants attended the laboratory after a ≥10 h overnight fast, having abstained from vigorous exercise for 48 h prior. A baseline muscle biopsy was sampled at rest, and participants subsequently ingested their allocated nutrient or placebo drink. Forty-five minutes post feeding, participants commenced SIT on a Wingate cycle ergometer (Monark 894E, Sweden). SIT comprised a standardized 5-min warm up of unloaded cycling at 60–70 rpm, followed by 4 × 30 s “all out” cycle sprints against a resistance equivalent to 7.5% BM, interspersed with 4-min active recovery periods. This was followed by a 3-min cool down period of unloaded cycling at 60–70 rpm. A second muscle biopsy was obtained 3-h post exercise, on the basis that our primary transcriptomic measure *PGC-1α*, among other targets, peaks post exercise at this time point (51). Between the postexercise recovery period and second biopsy, participants were not permitted any food or drink except for consuming water ad libitum. Serum blood sampling was completed before and after exercise at the following time points: baseline (Rest), immediately before exercise (Pre-Ex) (45-min post-drink ingestion), immediately (0 h), and 1-h postexercise (1 h).

Participants completed the remaining eight SIT sessions over a 3-wk period with 48–72 h rest between each exercise session (with training typically occurring on Mondays, Wednesdays, and Fridays). SIT consisted of 4–6 (4 sprints in *sessions 1*–*3*, 5 sprints in *sessions 4*–*6*, and 6 sprints in *sessions 7*–*9*) “all out” Wingate cycle sprints per session, using the same warm-up, cool-down, and recovery periods as on *day 1*. Participants refrained from completing any other forms of exercise apart from what was prescribed in the intervention. Preexercise nutritional conditions for these sessions were identical to *day 1*. Resting muscle biopsies and serum blood samples were obtained 48–72 h following *exercise session 9* for comparison with baseline.

### Performance Testing

Performance testing was completed at baseline, >48 h before *exercise session 1*, and 72–96 h following *exercise session 9*. Participants attended the laboratory >2 h after a self-selected preexercise meal (which was recorded and repeated in an identical manner for postintervention performance testing) and having refrained from caffeine intake on that day. Performance testing comprised in order as follows: *1*) a 30-s Wingate test and *2*) a 20-min cycling performance test, with 15 min of rest interspersed between performance tests. Post SIT performance testing was repeated by participants in an identical manner to baseline, 72–96 h following *exercise session 9*.

### Wingate Anaerobic Performance Test

Participants completed a single 30-s “all out” cycle sprinting against a resistance equivalent to 7.5% BM, with warm-up and cool-down periods the same as described on *day 1* of the exercise intervention. Performance was determined by mean power output and fatigue index.

### Aerobic Performance Test

After 15 min of seated rest, participants completed a 20-min cycling performance test (SRM, Germany). For this test, the ergometer was set in isokinetic mode where cadence was fixed at 85 rpm and power output was variable. Participants were instructed to exert maximal effort throughout the test, and standardized feedback and encouragement were provided regularly throughout the test. RPE and HR were monitored throughout the test and recorded at 5 min intervals. Performance was determined by mean power output achieved over the 20-min bout.

### Nutritional Intervention

All supplement and placebo drinks were matched for appearance, texture, and flavor (vanilla) and were presented to participants in a black, nontransparent container. Both protein drinks were isocaloric and isonitrogenous, while the placebo drink comprised of 0.33 g·kg^−1^ BM of a very low calorie (0.24 kcal·g^−1^), artificially flavored, and textured drink (85.3% Erythritol, Carbery Food Ingredients Ltd., Ireland), which acted as the fasted arm of the study. For both WPH and WPC groups, 0.33 g·kg^−1^ BM protein of a novel hydrolyzed WPH (Optipep4Power, Carbery Food Ingredients Ltd.) or WPC (Carbelac, Carbery Food Ingredients Ltd., Ireland) was provided, respectively. Dosages of protein drinks were selected to align with doses known to maximize postexercise MPS rates while minimizing amino acid oxidation rates (52), while the timing of protein intake was designed to align with the peak in circulating amino acid concentrations based on previous bioavailability data from our laboratory (53). Across the 241 exercise sessions completed as part of this study, we observed a total of 10 incidences of gastrointestinal distress. A summary of the amino acid profiles of both WPH and WPC products used in this study are presented in Table 1. All drinks were ingested 45 min preexercise, were food grade, and safe for human consumption. For *exercise sessions 2–9*, participants were also provided with a standardized postexercise snack (2.43 kcal·kg^−1^ BM, 0.25 g·kg^−1^ BM protein, 0.24 g·kg^−1^ BM CHO, 0.05 g·kg^−1^ BM fat), which they were instructed to consume not before 30-min after each exercise session.

**Table 1.** Amino acid profiles of WPH and WPC products used in experimental trials

	WPH (g·100 g^−1^ Protein)	WPC (g·100 g^−1^ Protein)
Leucine	10.6	11.5
Isoleucine	6.5	6.2
Valine	6.0	6.3
Aspartic acid	11.3	11.7
Glutamic acid	17.8	18.8
Serine	5.9	5.8
Glycine	1.9	2.1
Histidine	1.7	2.0
Arginine	2.3	3.0
Threonine	7.6	8.0
Alanine	5.4	5.3
Proline	6.0	6.2
Tyrosine	3.0	3.3
Methionine	2.2	2.0
Cysteine	2.4	2.6
Phenylalanine	3.0	3.5
Lysine	10.3	10.0
Tryptophan	1.9	1.5

WPC, whey protein concentrate; WPH, whey protein hydrolysate.

### Dietary Controls

Participants refrained from caffeine and alcohol intake for 12 h and 24 h, respectively, before each laboratory visit. Participants were instructed to maintain their habitual dietary intake for the duration of the intervention. A 7-day weighed food diary was completed by participants to characterize individual habitual dietary intakes among study participants before the intervention.

### Blood Sampling

Venous blood samples were collected and prepared in Serum Monovette tubes (Sarstedt, Wexford, Ireland) according to manufacturer’s instructions at the aforementioned time points. Serum aliquots were subsequently stored at −80°C for subsequent analysis.

### Muscle Biopsy Sampling

Muscle biopsy samples were obtained from m. vastus lateralis at baseline, 3-h *postexercise session 1*, and 48–72 h following the completion of *exercise session 9* using microbiopsy technique (54) (Medax Bio-feather, San Possidonio, Italy) under local anesthetic (1% lidocaine). Excess blood was quickly cleaned from all muscle samples using sterile gauze, which were dissected free of any visible fat and connective tissue. One piece of muscle sample was immediately snap-frozen in liquid nitrogen, designated for RNA analyses. Another, designated for protein expression and enzymatic activity analyses, was washed in Dulbecco’s PBS solution (Promo Cell; Heidelberg, Germany), and subsequently snap-frozen in liquid nitrogen. Samples were immediately stored at −80°C for further analyses.

### Multiplex PCR Analyses

A custom-designed multiplex gene expression (GeXP) assay using the GenomeLab Genetic Analysis System, the hMitoplex, was developed and used to evaluate mRNA expression of 20 genes implicated as regulators of mitochondrial biogenesis, substrate oxidation, and NAD^+^ biosynthetic activity in human skeletal muscle. Key methodological steps for this assay are published in greater detail elsewhere (55). Three reference genes were assessed for stable expression and UBE2D2 subsequently used to measure relative gene expression, having been confirmed as most stable across time points.

### Serum Metabolomics

A targeted quantitative metabolomics approach (TMIC Prime) was used to analyze samples using a combination of direct injection mass spectrometry with a reverse-phase liquid chromatography-tandem mass spectrometry (LC-MS/MS) custom TMIC Prime (The Metabolomics Innovation Center, University of Alberta, Edmonton, AB, Canada) assay (56, 57). Free fatty acids (FFAs) were analyzed using a previously described LC-MS/MS method (58) with some modifications. The custom-designed assays, in combination with an ABSciex 4000 Qtrap tandem mass spectrometry instrument (Applied Biosystems/MDS Analytical Technologies, Foster City, CA) equipped with an Agilent 1260 series UHPLC system (Agilent Technologies, Palo Alto, CA), were used for the targeted identification and quantification of 173 different endogenous metabolites including amino acids, acylcarnitines, organic acids, biogenic amines and derivatives, uremic toxins, glycerophospholipids, sphingolipids, and sugars (56, 57). The samples were delivered to the mass spectrometer by a LC method followed by a direct injection method. Data analysis was completed using Analyst 1.6.2 (Sciex, Framingham, MA).

### Protein Extraction

Muscle biopsy samples collected and prepared for Western blot and enzymatic activity analyses were homogenized using an ice-cold extraction buffer (1 M Tris pH 7.4, 0.5 M EDTA, 0.5 M Na_4_P_2_O_7_, 1 M NaCl, 1 M NaF, 10 mM nicotinamide, 1 mM PMSF, 1 mM Pepstatin A, 1 mM Na_3_VO_4_, 1 µM trichostatin A, Aprotinin 0.001%). Samples were subsequently centrifuged at 11,000 *g* for 10 min at 4°C, the supernatant removed, and protein concentration quantified using the method by Bradford (59).

### Citrate Synthase and β-HAD Activity Assays

Protein homogenates were analyzed to determine the maximal activity of citrate synthase (CS) and 3-hydroxyacyl-CoA dehydrogenase (β-HAD) on a spectrophotometer (BioTek Synergy HT, BioTek Instruments, Winooski, VT) using commercially available assay kits (CS Assay Kit No. CS0720, Sigma-Aldrich, UK) and reagents (50 mM Tris-HCl, 1 mM EDTA, 0.2% Triton-X-100, 5 mM NADH, 5 mM acetoacetyl CoA), according to manufacturer’s instructions and as described previously (60).

### Western Blotting

For Western blot analyses, samples were denatured before 20–40 μg were loaded on 4%–15% gradient SDS-PAGE precast gels (Mini-Protean TGX Stain-free, Bio-Rad, UK) for separation by gel electrophoresis. Following electrophoresis, gels were activated using UV-induced stain-free technology that activates tryptophan residues on the gel to determine total protein content (UVITEC Cambridge Imaging system UVITEC, Cambridge, UK). Separated proteins were transferred onto a 0.2-μM nitrocellulose membrane using semi-dry transfer technique and blocked in 5% skim milk powder in Tris-buffered saline (TBS) for 1 h at room temperature. Membranes were incubated at 4°C overnight in 5% bovine serum albumin (BSA) diluted in TBS and 0.1% tween-20 (TBST) with primary antibody solutions. The following primary antibodies and dilutions were used: Pan-acetylation (1:1,000; ab21623) and ac-MnSOD^K122^ (1:500; ab214675) antibodies were purchased from abcam; PARP1 (1:500; 9542), p53 (1:500; 2527), and ac-p53^K382^ (1:500; 2570) antibodies were purchased from Cell Signalling Technology; MnSOD (1:1,000; 06-984) and poly-ADP-ribosylation (1:500; MABE1031) antibodies were purchased from Merck Millipore. Expression of target proteins were normalized to total protein measurements made using stain-free technology. Secondary antibody green rabbit (1:10,000, 926-32211 IRDye 800 CW goat anti-rabbit IgG, LI-COR Biosciences UK Ltd., UK) and green mouse (1:7,500, 926-32210 IRDye 800 CW goat anti-mouse IgG, LI-COR Biosciences UK Ltd., UK) incubations were performed for 1 h at room temperature before imaging. Four TBST wash steps (4 × 5 min) were included following both primary and secondary antibody incubation steps.

### Statistical Analyses

Statistical analyses were performed using Statistical Package for the Social Sciences Version 26 (IBM Corp., NY) and RStudio version 1.2.5019 (RStudio, Boston, MA). For the acute study, total area under the curve (tAUC) for serum metabolites was calculated using the trapezoidal method. Differences in tAUC for serum metabolites, changes in gene expression, protein expression, protein acetylation, and poly-ADP-ribosylation status were all evaluated using a one-way analysis of variance (ANOVA), with Tukey’s post hoc multiple comparisons to identify differences between individual nutritional groups. Within-groups effects were evaluated using paired *t* tests of pre- and post-acute SIT data. Acute serum metabolomic data were also evaluated using a two-way analysis of covariance (ANCOVA), using baseline metabolite concentrations as a covariate to adjust for potential differences between groups at baseline, and a Bonferroni multiple-comparison correction was included.

For the chronic study, changes from baseline in gene expression, protein expression, protein acetylation, poly-ADP-ribosylation status, mitochondrial enzymatic activity, metabolite concentrations, and aerobic and anaerobic exercise performance were evaluated using a one-way ANOVA, with Tukey’s post hoc multiple comparisons to identify differences between individual nutritional groups. Within-groups effects were evaluated using paired *t* tests of pre- and postchronic SIT data. Multivariate data analyses [principal component analysis (PCA)] were conducted on serum metabolomic and muscle gene expression data at each time point to evaluate potential differences in the global metabolite and gene expression profiles between nutritional conditions. Partial least squares-discriminant analysis (PLS-DA) was carried out on the metabolomics data using the “ropls” package. Scores plots were created to visualize the PLS-DA models, and loadings provided information on the contribution of individual metabolites to differences between groups. The variable importance in the projection (VIP) was calculated to rank metabolites contribution to the classification of samples. Metabolites with VIP values >1.5 were considered most important in discriminating between groups. The goodness of the model was analyzed using both the *R*^2^ and *Q*^2^ parameters. PLS-DA models were validated by randomly splitting the data into training (55%) and test (45%) sets, this process was repeated 100 times and the predictive performance of the test sets of those repeats were recorded and averaged as the final estimation of the generalization performance of the model. Data are presented as means ± standard deviation (SD) unless otherwise stated, where *P* ≤ 0.05 indicates statistical significance. Effect sizes, where reported, are interpreted according to Cohen (61) where *D* = 0.2 (small); 0.5 (medium); 0.8 (large).

## RESULTS

### Baseline Characteristics

Summary baseline participant characteristics for each nutritional group are presented in Table 2. There were no significant between-groups differences in age (*P* = 0.512), fat mass index (*P* = 0.432), or V̇o_2max_ (*P* = 0.362). In addition, data from food diary analyses indicated that there were no significant differences in habitual energy (*P* = 0.523), protein (*P* = 0.652), CHO (*P* = 0.711), and fat (*P* = 0.583) intake between experimental groups.

**Table 2.** Summary baseline characteristics of study participants

Group	Age (y)	Fat Mass Index (kg·m^−2^)	V̇o_2max_ (mL·kg^−1^·min^−1^)
FAST	26.1 ± 5.1	4.4 ± 1.4	42.5 ± 4.4
WPC	23.6 ± 4.0	5.2 ± 1.8	40.0 ± 6.6
WPH	25.6 ± 4.5	5.3 ± 1.8	43.7 ± 3.7

Data are presented as means ± SD. FAST, fasted; WPC, whey protein concentrate; WPH, whey protein hydrolysate.

### Bioavailability of Amino Acids

Table 3 presents findings for the serum concentrations of amino acids at Rest, Pre-Ex, 0 h, and 1 h time points, as well as tAUC from the acute SIT trial. For the acute metabolomic analyses, an overall effect of protein was observed compared with FAST for increasing concentrations of 17 amino acids, as well as total branch chain amino acids (BCAA), essential amino acids (EAA), amino acids (AA), and non-essential amino acids (NEAA) (all *P* < 0.05). Supply of either WPH or WPC preexercise significantly elevated the overall amino acid profile during and up to an hour post exercise compared with FAST conditions, with a greater supply of threonine, glycine, alanine, asparagine, ornithine, total AAs, and total NEAAs provided by preexercise WPH ingestion compared with WPC, which was significantly greater than FAST (all *P* < 0.05).

**Table 3.** Serum amino acid concentrations (measured in μM) in WPH (n = 9), WPC (n = 9, n = 8 at Pre-Ex), and FAST (n = 9, n = 8 at 1 h post) groups at rest, Pre-Ex, 0 h, and 1 h time points, as well as associated tAUC values

Amino Acid	Group	Rest, μM	Pre-Ex, μM	0 h, μM	1 h, μM	tAUC, μM
Leucine^a^^,^^b^	WPH	159 ± 33	517 ± 173	321 ± 77	261 ± 80	40,222 ± 6,683
	WPC	164 ± 41	423 ± 108	366 ± 140	209 ± 66	37,457 ± 8,331
	FAST	176 ± 42	160 ± 32	140 ± 30	133 ± 13	18,158 ± 3,276
Isoleucine^a^^,^^b^	WPH	74 ± 24	177 ± 27	135 ± 23	118 ± 23	16,019 ± 1,793
	WPC	72 ± 17	168 ± 28	143 ± 35	95 ± 27	15,252 ± 2,417
	FAST	77 ± 23	72 ± 21	64 ± 16	61 ± 12	8,169 ± 2,051
Valine^a^^,^^b^	WPH	271 ± 39	447 ± 58	400 ± 34	352 ± 44	46,249 ± 3,279
	WPC	290 ± 59	430 ± 66	431 ± 95	321 ± 60	46,508 ± 7,780
	FAST	279 ± 45	263 ± 51	259 ± 47	249 ± 28	31,725 ± 5,116
Arginine^a^	WPH	109 ± 29	153 ± 25	130 ± 29	119 ± 20	15,855 ± 2,918
	WPC	105 ± 19	143 ± 28	130 ± 23	108 ± 17	15,075 ± 2,238
	FAST	103 ± 22	101 ± 26	99 ± 24	98 ± 21	12,171 ± 2,729
Histidine^a^^,^^b^	WPH	86 ± 9	105 ± 14	98 ± 16	93 ± 14	11,812 ± 1,575
	WPC	86 ± 12	103 ± 11	101 ± 16	89 ± 14	11,673 ± 1,531
	FAST	91 ± 11	89 ± 9	91 ± 8	95 ± 8	11,126 ± 1,062
Lysine^a^^,^^b^	WPH	188 ± 35	361 ± 71	262 ± 44	258 ± 25	33,425 ± 3,999
	WPC	192 ± 15	354 ± 69	299 ± 64	238 ± 47	33,819 ± 5,187
	FAST	212 ± 53	210 ± 61	190 ± 59	213 ± 46	24,754 ± 6,686
Methionine^a^^,^^b^	WPH	28 ± 8	51 ± 5	40 ± 5	38 ± 6	4,951 ± 530
	WPC	26 ± 4	45 ± 9	39 ± 7	31 ± 5	4,417 ± 585
	FAST	29 ± 7	28 ± 6	26 ± 5	28 ± 5	3,304 ± 637
Phenylalanine^a^^,^^b^	WPH	67 ± 12	87 ± 10	72 ± 9	69 ± 6	9,058 ± 909
	WPC	71 ± 9	86 ± 9	78 ± 11	70 ± 10	9,422 ± 1,067
	FAST	77 ± 15	72 ± 13	69 ± 13	71 ± 10	8,680 ± 1,528
Threonine^a^^,^^b^^,^^c^	WPH	118 ± 18	194 ± 25	176 ± 29	165 ± 22	20,582 ± 2,223
	WPC	107 ± 10	173 ± 22	156 ± 22	132 ± 24	17,631 ± 2,048
	FAST	108 ± 15	105 ± 15	95 ± 15	106 ± 13	12,395 ± 1,634
Tryptophan^a^^,^^b^	WPH	68 ± 8	104 ± 16	74 ± 6	84 ± 10	10,116 ± 830
	WPC	68 ± 8	103 ± 13	82 ± 17	82 ± 13	10,300 ± 1,268
	FAST	75 ± 6	70 ± 7	53 ± 8	63 ± 6	7,695 ± 540
Aspartic acid^a^	WPH	15 ± 5	22 ± 11	16 ± 4	16 ± 6	2,114 ± 521
	WPC	15 ± 3	16 ± 5	18 ± 4	16 ± 8	2,010 ± 511
	FAST	16 ± 4	11 ± 4	13 ± 4	13 ± 3	1,595 ± 339
Glutamic acid^a^^,^^b^	WPH	37 ± 8	63 ± 29	57 ± 8	45 ± 13	6,395 ± 1,074
	WPC	32 ± 11	47 ± 20	63 ± 23	37 ± 10	5,846 ± 1,698
	FAST	36 ± 10	31 ± 8	40 ± 9	33 ± 9	4,357 ± 616
Serine^a^	WPH	121 ± 13	180 ± 34	144 ± 27	136 ± 27	18,021 ± 2,635
	WPC	117 ± 8	153 ± 21	136 ± 20	120 ± 14	16,185 ± 1,682
	FAST	134 ± 21	129 ± 21	108 ± 22	124 ± 24	14,689 ± 2,635
Glycine^a^^,^^c^	WPH	221 ± 31	258 ± 35	211 ± 42	216 ± 45	27,541 ± 4,435
	WPC	199 ± 17	216 ± 24	184 ± 15	179 ± 16	23,465 ± 1,815
	FAST	233 ± 45	227 ± 45	198 ± 24	222 ± 31	26,326 ± 3,984
Alanine^a^^,^^c^	WPH	373 ± 87	527 ± 108	764 ± 136	652 ± 106	74,669 ± 10,882
	WPC	327 ± 55	416 ± 59	657 ± 67	523 ± 55	62,334 ± 5,569
	FAST	336 ± 81	337 ± 89	500 ± 106	550 ± 88	54,149 ± 10,328
Proline^a^	WPH	183 ± 39	284 ± 66	280 ± 68	281 ± 57	32,338 ± 6,975
	WPC	171 ± 53	256 ± 55	249 ± 53	229 ± 53	28,091 ± 6,342
	FAST	184 ± 48	180 ± 49	169 ± 42	218 ± 52	22,423 ± 5,890
Tyrosine^a^^,^^b^	WPH	69 ± 17	98 ± 17	77 ± 15	80 ± 16	9,957 ± 1,760
	WPC	67 ± 11	95 ± 12	78 ± 12	73 ± 11	9,615 ± 1,143
	FAST	68 ± 11	66 ± 12	51 ± 9	58 ± 9	7,213 ± 1,134
Glutamine^a^	WPH	551 ± 46	660 ± 67	633 ± 96	676 ± 89	77,711 ± 7,799
	WPC	549 ± 43	643 ± 60	644 ± 47	623 ± 62	75,861 ± 4,809
	FAST	623 ± 49	620 ± 56	616 ± 77	692 ± 54	77,080 ± 7,186
Asparagine^a^^,^^b^^,^^c^	WPH	47 ± 11	80 ± 14	71 ± 15	62 ± 7	8,204 ± 1,293
	WPC	43 ± 3	68 ± 10	64 ± 8	48 ± 8	6,960 ± 705
	FAST	47 ± 4	46 ± 3	47 ± 4	48 ± 4	5,686 ± 412
Total BCAAs^a^^,^^b^	WPH	505 ± 92	1,141 ± 244	857 ± 122	730 ± 139	102,489 ± 10,808
	WPC	526 ± 113	1,021 ± 190	940 ± 263	625 ± 149	99,217 ± 18,004
	FAST	532 ± 108	495 ± 101	463 ± 87	442 ± 50	58,052 ± 10,108
Total EAAs^a^^,b^	WPH	1,060 ± 140	2,043 ± 339	1,580 ± 141	1,438 ± 170	192,433 ± 11,631
	WPC	1,076 ± 140	1,885 ± 280	1,694 ± 361	1,267 ± 215	186,479 ± 23,753
	FAST	1,125 ± 185	1,069 ± 190	987 ± 177	1,018 ± 109	126,006 ± 19,811
Total NEAAs^a^^,^^c^	WPH	1,885 ± 218	2,506 ± 257	2,555 ± 384	2,446 ± 269	293,528 ± 30,428
	WPC	1,758 ± 135	2,201 ± 199	2,368 ± 187	2,084 ± 162	262,612 ± 14,899
	FAST	1,936 ± 226	1,895 ± 254	1,971 ± 207	2,192 ± 195	242,611 ± 26,200
Total AAs^a^^,^^b^	WPH	2,945 ± 335	4,549 ± 512	4,135 ± 489	3,884 ± 415	485,961 ± 39,417
	WPC	2,834 ± 224	4,086 ± 377	4,063 ± 503	3,350 ± 298	449,092 ± 28,908
	FAST	3,060 ± 345	2,964 ± 377	3,210 ± 260	3,210 ± 260	368,616 ± 3,9810

Data are presented as means ± SD. FAST, fasted; Pre-Ex, prior to exercise; tAUC, total area under the curve; WPC, whey protein concentrate; WPH, whey protein hydrolysate; BCAA, branch chain amino acids; EAA, essential amino acids; NEAA; non-essential amino acids; AA, amino acids.

^a^ Significant difference in amino acid concentrations (tAUC) between WPH and FAST groups (*P* < 0.05); ^b^significant difference in amino acid concentrations (tAUC) between WPC and FAST groups (*P* < 0.05); ^c^significant difference in amino acid concentrations (tAUC) between WPH and WPC groups (*P* < 0.05).

### Serum Metabolites

**Figure 2.**
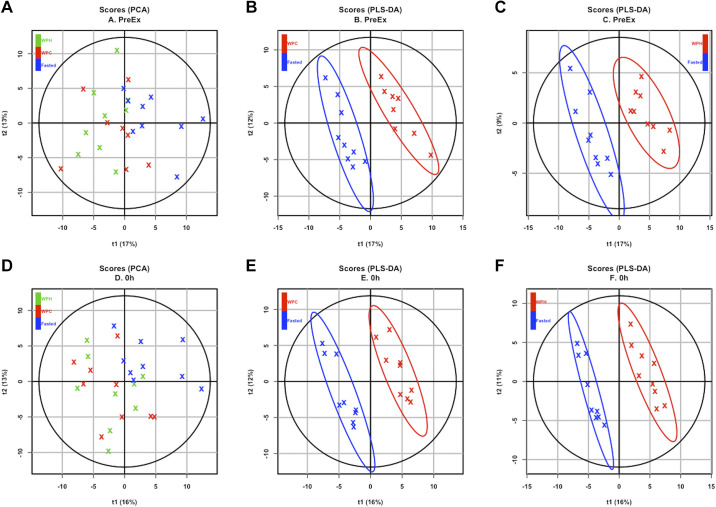
*A*: principal component analysis (PCA) plot showing principal component one (PC1, 17%) versus principal component two (PC2, 13%) of liquid chromatography-tandem mass spectrometry (LC-MS) metabolomics data from fasted (FAST, blue), whey protein concentrate (WPC, red), and whey protein hydrolysate (WPH, green) groups at preexercise time point. *B*: partial least squares-discriminant analysis (PLS-DA) scores plots (*R*^2^ = 0.43 and *Q*^2^ = 0.73) of metabolites from WPC and fasted groups preexercise. *C*: PLS-DA scores plots (*R*^2^ = 0.39 and *Q*^2^ = 0.73) of metabolites from WPH and fasted groups preexercise. *D*: PCA plot showing principal component one (PC1, 16%) versus principal component two (PC2, 13%) of LC-MS metabolomics data from WPC, WPH, and fasted groups at 0 h time point. *E*: PLS-DA scores plots (*R*^2^ = 0.41 and *Q*^2^ = 0.70) of metabolites from WPC and fasted groups at 0 h. *F*: PLS-DA scores plots (*R*^2^ = 0.39 and *Q*^2^ = 0.74) of metabolites from WPH and fasted groups at 0 h.

Multivariate analysis using PCA showed that at rest there were no differences between nutritional groups in the overall global metabolite profile (data not shown). For the Pre-Ex (45-min post-drink ingestion) and 0 h time points, PCA showed discrimination between nutritional groups (Fig*. 2, A* and *D*), whereas at the 1 h and post-training time points, no clear discrimination in the overall metabolite profile was observed between nutritional groups (data not shown).

Pairwise PLS-DA models at Pre-Ex (Fig. 2, *B* and *C*) and 0 h time points (Fig. 2, *E* and *F*) showed discrimination between WPH and FAST and WPC and FAST groups. Analytes with a VIP score of >1.5 at Pre-Ex and 0 h time points for comparisons between groups showing discrimination in global metabolite profiles are summarized in Supplemental File S1 (see https://doi.org/10.6084/m9.figshare.15029316.v1), presenting discriminating metabolites (VIP > 1.5) for each PLS-DA model in addition to univariate one-way ANOVA and two-way ANCOVA. Acute profiles of three biogenic amines, four acylcarnitines, and two organic acids were different in protein-fed versus FAST conditions (all *P* < 0.05). Serum α-aminoadipic acid, kynurenine, and *trans*-hydroxyproline were higher after protein ingestion (all *P* < 0.05), with a greater increase in *trans*-hydroxyproline in the WPH group compared with WPC (*P* = 0.002). Protein supplementation also increased the serum concentration of the acylcarnitines C3, C4, C4OH, and C5 (*P* < 0.05), whereas circulating C10:1 was significantly elevated in FAST compared with protein feeding conditions (*P* < 0.05). Indole acetic acid and β-hydroxybutyric acid concentrations were augmented with protein supplementation compared with FAST (*P* < 0.05). Two amino acids, two glycerophospholipids, one acylcarnitine, three organic acids, and total NEAAs were different between nutritional groups after 3 wk of training. However, only C4:1 which was lower in the WPH group was different in both FAST (*P* = 0.003) and WPC (*P* = 0.013) groups.

### Multiplex Gene Expression

**Figure 3.**
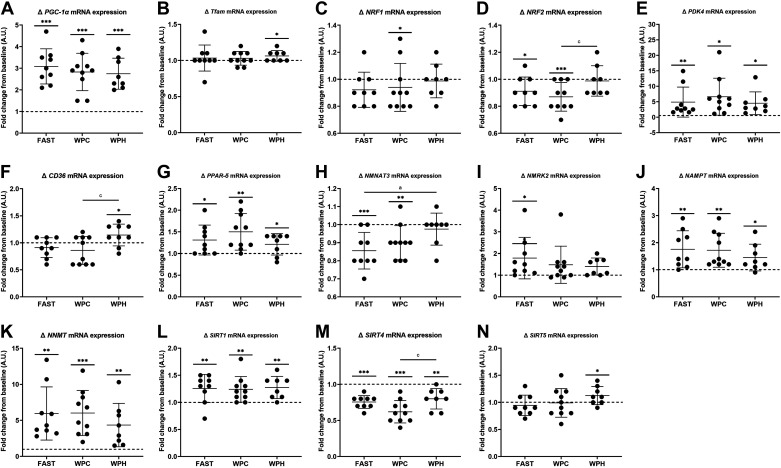
*A*–*N*: exercise-induced gene expression changes observed in response to acute sprint interval training (SIT) in fasted (FAST, *n* = 9 participants), whey protein concentrate (WPC, *n* = 10), and whey protein hydrolysate (WPH, *n* = 8) nutritional groups. Data are presented as means ± SD. *P < 0.05 within-groups; **P < 0.01 within-groups; ***P < 0.001 within-groups. ^a^Significant difference in exercise-induced gene expression between WPH and FAST groups; ^c^significant difference in exercise-induced gene expression between WPH and WPC groups. Dashed line indicates relative baseline value for each participant.

PCA indicated that no patterns of gene expression were associated with the different nutritional interventions at baseline, 3-h postexercise, or post-training time points (Supplemental File S1). Following an acute bout of SIT, altered *PGC-1α*, *Tfam*, *NRF1*, *NRF2*, *PDK4*, *PPAR-δ, SIRT1*, *SIRT4*, *NMNAT3*, *NMRK2*, *NAMPT*, and *NNMT* mRNA expression was observed 3-h postexercise (all *P* < 0.05, Fig. 3, *A*–*N*). The remaining genes were unchanged from baseline (*P* > 0.05).

For the exercise-induced change in gene expression, no between-groups differences were detected for *PGC-1α*, *Tfam*, *NRF1*, *PDK4*, *PPAR-δ, SIRT1*, *NAMPT*, *NMRK2*, and *NNMT*. One-way ANOVA identified significant between-groups differences for exercise-induced *NRF2* (*P* = 0.038)*, NMNAT3* (*P* = 0.025), *CD36* (*P* = 0.032), and *SIRT4* (*P* = 0.025) mRNA expression. *NRF2* mRNA expression decreased post exercise in FAST (*P* = 0.014) and WPC (*P* = 0.001), but not WPH conditions (*P* = 0.445). Post hoc analyses revealed differences between WPH and WPC conditions for the change in *NRF2* expression (*P* = 0.033, *D* = 1.3). *NMNAT3* mRNA expression was decreased post exercise in FAST (*P* = 0.001) and WPC (*P* = 0.002), but not in WPH conditions (*P* = 0.311), with a significant difference in exercise-induced *NMNAT3* expression between WPH and FAST conditions (*P* = 0.022, *D* = 1.4). *CD36* mRNA expression was augmented post exercise in the WPH group (*P* = 0.048), with post hoc testing showing differences between WPH and WPC conditions for exercise-induced *CD36* mRNA expression (*P* = 0.031, *D* = 1.2). For *SIRT4*, mRNA expression was downregulated 3-h post exercise in all nutritional conditions (*P* < 0.0001 for FAST and WPC, *P* = 0.002 for WPH), with post hoc analyses identifying differences between WPH and WPC conditions (*P* = 0.027, *D* = 1.2).

**Figure 4.**
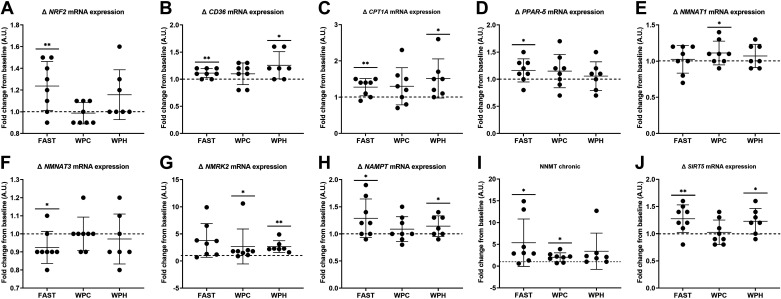
*A*–*J*: training-induced resting gene expression changes observed in response to 3 wk of sprint interval training (SIT) in fasted (FAST, *n* = 8), whey protein concentrate (WPC, *n* = 8), and whey protein hydrolysate (WPH, *n* = 7) nutritional groups. Data are presented as means ± SD. *P < 0.05 within-groups; **P < 0.01 within-groups; dashed line indicates relative baseline value for each participant.

Following 3 wk of SIT, resting mRNA expression levels of various hMitoplex genes were differentially regulated. Training-induced changes in *NRF2*, *PDK4*, *CD36*, *CPT1A*, *SIRT3*, *SIRT5*, *NMNAT3*, *NMRK2*, *NAMPT*, and *NNMT* expression were observed (*P* < 0.05, Fig. 4, *A*–*J*), with no effect of nutritional condition (*P* > 0.05, Fig. 4, *A*–*J*). The remaining targets were unchanged from baseline.

### Muscle Protein Expression, Acetylation, and ADP-Ribosylation Status

**Figure 5.**
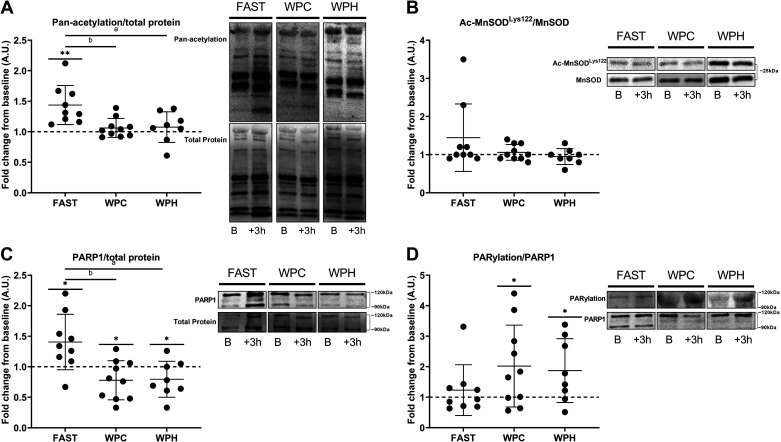
Changes in skeletal muscle MnSOD^Lys122^ acetylation (*A*), pan-acetylation (*B*), PARP1 protein content (*C*), and PARylated PARP1 (*D*) in response to acute sprint interval training (SIT) in fasted (FAST, *n* = 9 participants), whey protein concentrate (WPC, *n* = 10), and whey protein hydrolysate (WPH, *n* = 8) nutritional groups. B, baseline; +3 h, 3-h post exercise. Data are presented as means ± SD. *P < 0.05 within-groups; **P < 0.01 within-groups; ^a^significant difference in exercise-induced activity/expression between WPH and FAST groups; ^b^significant difference in exercise-induced activity/expression between WPC and FAST groups. Dashed line indicates relative baseline value for each participant.

**Figure 6.**
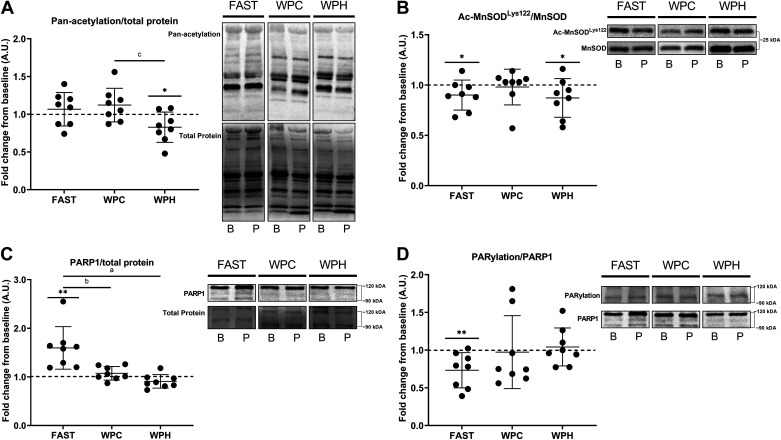
Changes in resting skeletal muscle MnSOD^Lys122^ acetylation (*A*), pan-acetylation (*B*), PARP1 protein content (*C*), and PARylated PARP1 (*D*) in response to 3 wk of sprint interval training (SIT) in fasted (FAST, *n* = 8 participants), whey protein concentrate (WPC, *n* = 8), and whey protein hydrolysate (WPH, *n* = 8) nutritional groups. B, baseline; P, post training. Data are presented as means ± SD. *P < 0.05 within-groups; **P < 0.01 within-groups; ^a^significant difference in training-induced activity/expression between WPH and FAST groups; ^b^significant difference in training-induced activity/expression between WPC and FAST groups; ^c^significant difference in training-induced activity/expression between WPH and WPC groups. Dashed line indicates relative baseline value for each participant.

In response to acute SIT, pan-acetylation of skeletal muscle was unchanged 3-h post exercise in WPC (*P* = 0.116) and WPH conditions (*P* = 0.205), but was increased in FAST (*P* = 0.002). One-way ANOVA indicated that exercise-induced pan-acetylation was significantly different between nutritional conditions (*P* = 0.005), with post hoc testing identifying differences between both WPH (*P* = 0.017, *D* = 1.3) and WPC (*P* = 0.008, *D* = 1.6) conditions compared with FAST (Fig. 5*A*). After 3 wk of training, resting muscle pan-acetylation was unchanged from baseline in both FAST (*P* = 0.212) and WPC (*P* = 0.083) conditions, but was decreased in WPH (*P* = 0.023). Training-induced changes in resting pan-acetylation status were significantly different between groups (*P* = 0.029, Fig. 6*A*), with post hoc tests showing differences between WPH and WPC conditions (*P* = 0.032, *D* = 1.4), and a trend for differences between WPH and FAST groups (*P* = 0.091, *D* = 1.1).

Following both acute and chronic SIT, acetylation of p53^Lys382^ was unchanged from baseline in all nutritional groups (*P* > 0.05), with one-way ANOVA indicating no between-groups differences in exercise- and training-induced p53^Lys382^ acetylation status (*P* > 0.05, data not shown). Acetylation of MnSOD^Lys122^ was not altered in any nutritional group following acute SIT (*P* > 0.05, Fig. 5*B*), with no between-groups difference in its exercise-induced acetylation (*P* = 0.143). Resting MnSOD^Lys122^ acetylation was decreased from baseline in FAST (*P* = 0.050) and WPH (*P* = 0.049) conditions following 3 wk of SIT, with no change in WPC (*P* = 0.376). Training-induced changes in acetylation of MnSOD^Lys122^ were not different between nutritional conditions (*P* = 0.445, Fig. 6*B*).

PARP1 protein expression was augmented following acute SIT in FAST conditions (*P* = 0.014), whereas it was decreased in both WPC (*P* = 0.029) and WPH (*P* = 0.044). Exercise-induced PARP1 expression was significantly different between nutritional conditions (*P* = 0.001), with post hoc testing demonstrating differences between both WPC (*P* = 0.003, *D* = 1.6) and WPH (*P* = 0.006, *D* = 1.6) protein feeding conditions when compared with FAST (Fig. 5*C*). PARylation of PARP1 increased 3-h post exercise in both WPC (*P* = 0.020) and WPH (*P* = 0.025) groups, with no change in fasted conditions (*P* = 0.214). Exercise-induced changes in PARylation of PARP1 were not different between nutritional groups (*P* = 0.286, Fig. 5*D*). Following 3 wk of SIT, resting PARP1 protein content was unchanged in WPC (*P* = 0.091) and WPH (*P* = 0.051) conditions and increased in the FAST group (*P* = 0.003). One-way ANOVA demonstrated that training-induced changes in PARP1 content differed between nutritional groups (*P* = 0.0002), with post hoc analyses revealing differences between both WPC (*P* = 0.003, *D* = 1.6) and WPH conditions (*P* = 0.0002, *D* = 2.1) compared with FAST (Fig. 6*C*). PARylation of PARP1 in resting skeletal muscle post-training was unchanged in WPC (*P* = 0.438) and WPH (*P* = 0.324) conditions, but decreased from baseline in the FAST group (*P* = 0.007). One-way ANOVA indicated that training-induced changes in PARylated PARP1 were not different between groups (*P* = 0.190, Fig. 6*D*).

### Muscle Enzymatic Activity

**Figure 7.**
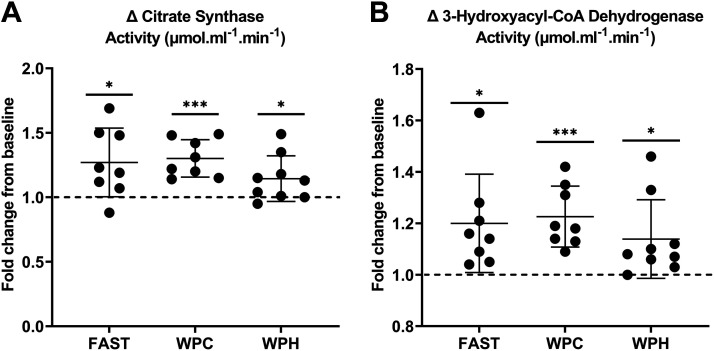
Change in citrate synthase (CS, *A*) and 3-hydroxyacyl-CoA dehydrogenase (β-HAD, *B*) activities observed in response to 3 wk of sprint interval training (SIT) in whey protein hydrolysate (WPH, *n* = 8 participants), whey protein concentrate (WPC, *n* = 9), and fasted (FAST, *n* = 8) nutritional groups. Data are presented as means ± SD. *P < 0.05 within-groups; ***P < 0.001 within-groups. Dashed line indicates relative baseline value for each participant.

Following 3 wk of SIT, maximal activities of CS and β-HAD increased in all nutritional groups (*P* < 0.05). No differences between FAST, WPC, and WPH groups were demonstrated for the change in CS (*P* = 0.254) and β-HAD (*P* = 0.544) activity (Fig. 7).

### Aerobic Exercise Performance

**Figure 8.**
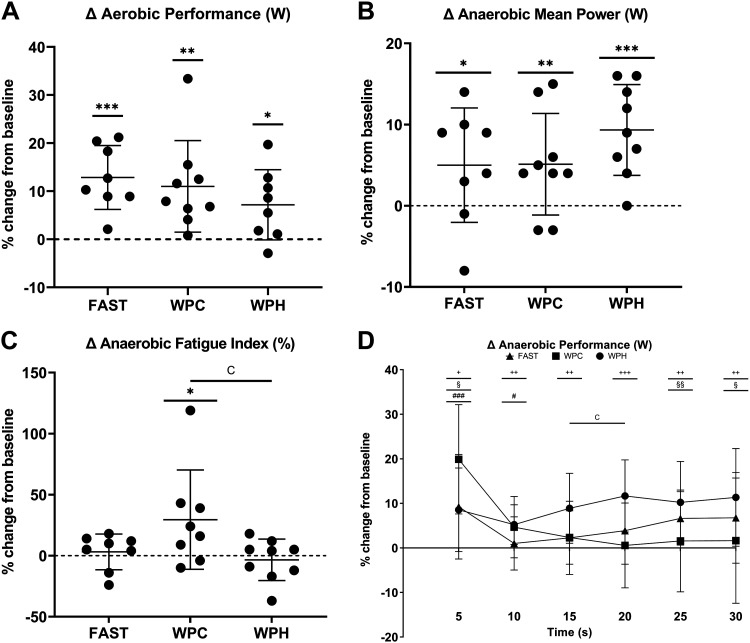
*A*–*D*: Change in aerobic and anaerobic performance measures observed in response to 3 wk of sprint interval training (SIT) in fasted (FAST, *n* = 8 participants), whey protein concentrate (WPC, *n* = 9, *n* = 8 for fatigue index), and whey protein hydrolysate (WPH, *n* = 9) nutritional groups as assessed by one-way ANOVA. The percentage change from baseline in aerobic mean power output (*A*), anaerobic mean power (*B*), anaerobic fatigue index (*C*), and temporal change in anaerobic performance (6 × 5 s) (*D*) are presented. Data are presented as means ± SD. In *A–C*, *P < 0.05 within-groups; **P < 0.01 within-groups; ***P < 0.001 within-groups. In D §*P* < 0.05, §§*P* < 0.01 within FAST group; #*P* < 0.05, ###*P* < 0.001 within WPC group; +*P* < 0.05, ++*P* < 0.01, +++*P* < 0.001 within WPH group. ^c^Significant difference in performance measure between WPH and WPC groups.

Power output during the aerobic performance test was augmented in all nutritional conditions following SIT (*P* < 0.05, Fig. 8*A*), with no differences in training-induced changes in aerobic performance (*P* = 0.365) between either WPH and FAST (*D* = 0.7), WPC and FAST (*D* = 0.3), or WPH and WPC groups (*D* = 0.4).

### Anaerobic Exercise Performance

Wingate mean power was increased following training in all nutritional groups (*P* < 0.05, Fig. 8*B*). Despite the observation of moderate-to-large effects showing a greater magnitude of mean power change in WPH compared with WPC (*D* = 0.7) and FAST (*D* = 0.7) conditions, no difference in training-induced Wingate mean power was observed between groups (*P* = 0.277). Wingate fatigue index was unchanged from baseline following SIT in FAST (*P* = 0.31), increased in WPC (*P* = 0.030), and tended to be decreased in WPH conditions (*P* = 0.074). One-way ANOVA demonstrated that training-induced changes in fatigue index were different between nutritional conditions (*P* = 0.045, Fig. 8*C*), with post hoc analyses identifying differences between WPH and WPC groups (*P* = 0.045, *D* = 0.5), indicating improved fatigue index in the WPH group. Figure 8*D* provides a profile of the temporal change in anaerobic power output stratified into individual 5-s periods, which identified a greater increase in training-induced power output in WPH compared with WPC groups for 16–20 s of the Wingate test (*P* = 0.027, *D* = 1.3).

## DISCUSSION

Manipulation of nutritional intake around acute exercise bouts, particularly training in the fasted or CHO-restricted state, has garnered interest as a strategy to augment postexercise metabolic adaptations (62). However, fasted exercise may induce a net negative muscle protein balance and consequent need for increased protein intakes (37). Previous research has evaluated divergent responses to fasted compared with CHO-fed endurance exercise (5, 10). To our knowledge, this is the first study investigating the effects of preexercise protein feeding on metabolic and performance adaptations to SIT. Our findings observed novel adaptations in response to acute and chronic SIT under fasted, WPC, and WPH nutritional conditions, specifically in relation to the serum metabolome, regulation of skeletal muscle mitochondrial adaptation, substrate oxidation, NAD^+^ biosynthetic capacity, mitochondrial enzymatic activity, protein activity (acetylation), DNA repair, and exercise performance. The implications of these findings suggest that while fasted SIT is a potent physiological stimulus, preexercise feeding of protein, particularly WPH, induces some novel benefits in terms of specific metabolic adaptations and some aspects of anaerobic performance.

### Metabolomics

Multivariate analysis highlighted differences in global metabolite profiles between fasted, WPC, and WPH conditions after feeding, immediately pre- and postexercise. This is unsurprising, particularly when comparing protein-fed conditions and fasting. The greatest differences were between the WPH and fasted conditions. The metabolites driving separation after feeding and preexercise reflect the composition of the protein drinks (e.g., higher BCAAs and EAAs) or the known metabolic signature of a fasted state (e.g., higher medium- and long-chain acylcarnitines, organic acids); whereas post-exercise we see differences in BCAAs, EAAs, and acylcarnitines.

Total BCAA and EAA profiles were comparable between WPH and WPC groups. Protein hydrolysates are more rapidly absorbed from the gut due to the enzymatic hydrolysis process, which can modify peptide chain length to more readily absorbed di- and tripeptides (48), in addition to producing bioactive peptides derived from intact protein sources that are associated with functional effects in vivo (63). EAAs primarily drive muscle protein synthesis (64, 65), evidence which should be factored into the interpretation of our findings concerning amino acid profiles in WPH and WPC groups. Findings of interest pertaining to divergences in amino acid profiles and their intermediates are for the EAA threonine, the NEAA glycine, and the nonproteinogenic amino acid and metabolic intermediate ornithine. Serum concentrations of these amino acids during acute SIT were greater in WPH conditions. Threonine exhibits bioactive anabolic properties in humans (66). Glycine proposedly enhances muscle protein mass in inflammatory conditions in animals (67), and protects muscle cells from atrophy via mTOR complex 1 (mTORC1) signaling in vitro (68). These potential implications for muscle anabolism or recovery require further research. Research proposes that ornithine exerts antifatigue effects via beneficial modulation of lipid metabolism during exercise (69), which is of interest given its elevation in WPH conditions in this study. This is additive to the currently available literature observing no inhibitory effect, and in some cases a benefit of protein ingestion on the augmented fatty acid oxidation, which is characteristic of fasted exercise (39–42). Our findings support this assertion along with the fact that preexercise protein ingestion can simultaneously ensure the delivery of adequate amino acid supply during SIT. These findings could be viewed as indicating greater bioactivity associated with the consumption of WPH, however, it is important to note these differences are small in magnitude.

Biogenic amines altered in response to exercise and nutritional conditions included α-aminoadipic acid, kynurenine, and *trans*-hydroxyproline. These were acutely increased in both protein feeding conditions, with further increases in *trans*-hydroxyproline observed in WPH conditions. The acute increases in kynurenine were expectedly higher in both WPH and WPC groups given the increased tryptophan supply and may be important in the context of NAD^+^ biosynthesis, since this pathway regulates the intracellular NAD^+^ supply (70). This finding highlights a benefit for pre-exercise protein supplementation in potentially augmenting NAD^+^ biosynthesis compared with the fasted state, while our collective findings support the hypothesis that protein ingestion does not blunt activity of this system in the same manner as CHO-fed conditions (19, 20, 22). α-Aminoadipic acid is a lysine metabolite which decreases fasting glucose concentrations and enhances insulin sensitivity in animals in vivo (71, 72). α-Aminoadipic acid suppresses protein degradation and autophagy in vitro, with concomitant upregulation of the Akt/mTOR signaling pathway (73). This evidence is of interest given the elevation of this metabolite in protein-fed conditions compared with the fasted state, potentially suggesting a mitigation of protein degradation in these nutritional groups. The observed difference in *trans*-hydroxyproline supply between nutritional conditions, with the greatest overall supply in the WPH group, followed by WPC, is another finding of interest. *Trans*-hydroxyproline exerts protective effects from oxidative stress and injury in vitro (74, 75), and beneficially regulates markers of cellular antioxidant activity (74–77). Such findings outline a potential antioxidant effect of protein supplementation compared with the fasted state, and in addition, a greater antioxidant effect of WPH compared with WPC. However, this remains as yet untested.

During and following acute SIT, protein feeding augmented concentrations of short-chain acylcarnitines C3, C4, C4OH, and C5 whereas the medium change acylcarnitine C10:1 was augmented in fasted conditions. Acylcarnitines are intermediate oxidative metabolites with functions in mitochondrial fatty acid transport and β-oxidation (78). Apart from their generation from the breakdown of glucose and fatty acids (79), the observed increase in short-chain acylcarnitine concentrations may be derived from BCAA metabolism, having been previously observed for C3 and C5 following whey protein ingestion (80). Metabolism of short-chain carnitines and the interactions of acetylcarnitine and acetyl-CoA via the enzyme carnitine acetyltransferase proposedly regulates pyruvate dehydrogenase complex activity, and in turn glucose oxidation (81). Medium-chain acylcarnitines are increased in response to endurance exercise and support palmitate oxidation (82). Our findings may indicate that in our initial exercise session, substrate supply in protein-fed conditions exceeded metabolic flux of specific mitochondrial enzymes, resulting in acyl-CoA accumulation and subsequent increases in acylcarnitine production (83). However, following short-term training we observe lower resting concentrations of C4:1 in the WPH group. This potentially reflects more efficient short-chain acylcarnitine utilization as a metabolic adaptation. The organic acids, indole acetic acid and β-hydroxybutyric acid, were augmented in the acute study in both protein-feeding conditions. Although these findings are interesting, little human research has investigated the effects of protein supplementation on the metabolism of these organic acids, thus it is difficult to discern the biological relevance of these findings.

### Muscle Gene Expression

PCA revealed that the overall gene expression patterns were not differentially regulated by nutritional condition either acutely or chronically. Acute SIT upregulated the expression of genes implicated in the regulation of mitochondrial biogenesis (*PGC-1α*, *SIRT1*), substrate oxidation (*PDK4*, *PPAR-δ)*, and *NAD^+^* biosynthesis (*NAMPT*, *NNMT*), independent of nutritional condition. These findings of an acute exercise effect on the regulation of these targets are evident in the extant literature (8, 21, 22, 84). However, findings for altered regulation following acute SIT are novel for *NRF1*, *PDK4*, *SIRT1*, *SIRT4*, *NMNAT3*, and *NAMPT*. Protein supplementation does not blunt exercise-induced AMPK phosphorylation and metabolic gene expression (41, 84), or increases in circulating FFAs and whole body lipid oxidation that are characteristic of fasted exercise (39). These findings support the hypothesis that protein supplementation does not inhibit key signaling intermediaries influenced by glucose and glycogen levels (15, 19, 20). This hypothesis is further bolstered by our metabolomics analysis, which did not observe major differences in the profile of metabolites expected to be typically elevated during fasted exercise, such as organic acids or acylcarnitines, with the sole exception being the medium chain acylcarnitine C:10:1, which was augmented in fasted conditions.

Exercise-induced expression of *CD36* was greater in WPH compared with WPC conditions. Available evidence regarding the efficacy of WPH and WPC supplementation to support muscle metabolic adaptations to SIT and endurance exercise is sparse. Other research has indicated that skeletal muscle CD36 primarily functions as a sarcolemmal FFA transporter (85, 86), with FFAs subsequently stored in skeletal muscle as intramuscular triglycerides (IMTGs) or synthesized into ATP via fatty acid oxidation (87, 88). *CD36* knockout (KO) mice exhibit reduced exercise, fatty acid transport, and oxidation capacities (87). The precise mechanism by which WPH may have augmented *CD36* expression remains unclear, as little research exists regarding the role of protein supplementation in regulating markers of skeletal muscle lipid metabolism. However, given the role of *CD36* in fatty acid transport and metabolism, its upregulation in WPH-fed conditions is intriguing. Previous studies have observed that exercise-induced increases in muscle acylcarnitines positively associate with the induction of several lipid metabolic genes (83), and although the mechanisms of cellular acylcarnitine transport remain unresolved it cannot be ruled out that *CD36* may be a potential player in this process (89). Between-groups effect sizes for the WPH group compared with WPC and fasted conditions were large, whereas the percentage differences between these groups ranged from 22% to 28%. For context, comparing these data with information from the MetaMEx skeletal muscle transcriptomic database, mean changes in response to acute aerobic exercise or HIIT at the same sampling time point used in this study were 10%–12% (90). Such information may suggest that these findings are biologically meaningful as well as statistically significant.

*SIRT4* inhibits mitochondrial fatty acid oxidation (91, 92), whereas *SIRT4* KO mice exhibit enhanced fatty acid oxidation rates and increased exercise capacities (93). In contrast to *CD36*, greater exercise-induced *SIRT4* downregulation was evident in the WPC compared with WPH group with a large between-groups effect. Although exercise-induced *SIRT4* downregulation was not significantly different between WPC and fasted conditions, a large between-groups effect size was observed. Although previous studies have evaluated *SIRT4* regulation in human whole blood in response to SIT (94), to our knowledge no research has determined exercise and nutritional factors on *SIRT4* mRNA expression in human skeletal muscle. Our evidence regarding *SIRT4* mRNA expression and protein feeding contrast with findings for *CD36* gene expression. The reason for this divergence is unclear, but suggests these targets are regulated independently. Further research is warranted to establish the divergent response of these genes involved in fatty acid metabolism in response to preexercise protein feeding.

### Muscle Signaling and Enzymatic Activity

Preexercise intake of either WPH or WPC appeared to prevent the exercise-induced increase in muscle pan-acetylation that was observed in fasted conditions following an acute bout of SIT. Furthermore, following 3 wk of training a decrease in resting pan-acetylation status was evident in the WPH condition, which was different from WPC, and tended to differ from fasted conditions with a large between-groups effect. Recent research has found no effect of acute endurance exercise on human skeletal muscle pan-acetylation status (95). Other authors observed an increase in the expression of SIRT deacetylases over accumulated bouts of HIIT (96), which are known to exert regulatory effects on mitochondrial fatty acid oxidation and other metabolic processes (24). Evidence indicates that almost all proteins involved in skeletal muscle contraction are acetylated, and the subcellular component where lysine acetylation is most prominent is the mitochondria, with 63% of proteins containing acetylation sites (25, 97). Lysine acetylation sites are found on proteins involved in all key mitochondrial enzymatic steps involved in the generation of ATP (97). Animals bred for high running capacity had significantly lower mitochondrial protein acetylation at rest and in response to exercise, with specific effects within the oxidative phosphorylation, TCA cycle, BCAA degradation, and fatty acid metabolism pathways, when compared with animals bred for low-running capacity (98). Deacetylation has been previously implicated in favorably mediating the transcription of genes regulating mitochondrial biogenesis and substrate metabolism (99–101), whereas hyperacetylation has been proposed to stimulate muscle proteolysis and atrophy (102). Thus, our findings indicating resting acetylation status is reduced in WPH conditions following 3 wk of SIT are of interest and add to the extant literature, potentially outlining a beneficial effect of WPH in stimulating deacetylation, which in turn may have other downstream effects in terms of muscle metabolic adaptation.

Acetylation of p53^Lys382^ and MnSOD^Lys122^, which are downstream targets of SIRT1 and SIRT3 deacetylase activity respectively, were also assessed in this study. Similar to other exercise trials (95), there was no effect of acute SIT on SIRT1 and SIRT3 activity. Following 3 wk of training, resting SIRT1 activity was unchanged, but acetylation of MnSOD^Lys122^ decreased in WPH and FAST conditions, indicating an increase in SIRT3 activity. Rodent studies have observed increased SIRT1 deacetylase activity following exercise, although ablation of SIRT1 appears to have little effect on mitochondrial adaptations and exercise capacity (103). Research on SIRT3 suggests that it can regulate mitochondrial ATP levels by deacetylating TCA cycle intermediaries, fatty acid oxidation promotors (104–106), and exert protective effects against oxidative stress by deacetylating antioxidant enzymes (107, 108). Given that training-induced changes in SIRT3 activity were not different between groups, the biological significance of these findings may be trivial.

We also measured acute and chronic changes in PARP1 protein and its PARylation, finding that following acute SIT PARP1 content increased post exercise in fasted conditions, but decreased in both protein feeding conditions. Although differences in exercise-induced PARP1 content were significant between protein-fed and fasted conditions, PARylation of PARP1 was not. Following 3 wk of SIT, training-induced changes in PARP1 content differed between fasted conditions where an increase from baseline was evident, compared with protein feeding conditions where no change occurred. Once again, training-induced changes in PARP1 PARylation were not different between nutritional groups. PARP1 is a major consumer of NAD^+^ and competes with SIRTs for its consumption, with its PARylation activity being increased in response to DNA damage (109). Genetic deletion of PARP1 enhances oxidative metabolism in mice (110), whereas other animal models have identified that pharmacological inhibition of PARP1 enhances intracellular NAD^+^ content, mitochondrial biogenesis, and exercise capacity (111, 112). PARP1 PARylation is an important post-translational modification with an important regulatory role in cell survival and autophagy, which requires significant NAD^+^ and ATP supply (113, 114). Overactivation of PARP1 by oxidative stress leads to its increased PARylation, depleting intracellular NAD^+^ levels and promoting cell death (115). Thus, glycolytic and TCA cycle activity, among other processes, will be compromised in the presence of significant PARP1 activity (113). Previous studies have found no effect of acute endurance exercise on PARP1 content or its PARylation in human skeletal muscle (95). Other research has observed that low physical activity levels and aging are associated with increased skeletal muscle PARP1 content (116). Our findings indicate an attenuating effect of both WPH and WPC supplementation on the increase in PARP1 protein content, which was observed in the fasted exercise condition following both acute and chronic SIT. The corresponding changes in PARP1 PARylation we observed appeared to be primarily driven by the underlying changes in PARP1 content, which may call into question the biological significance of these findings, particularly given the lack of between-groups differences in exercise- and training-induced PARP1 PARylation. However, since PARP1 conducts over 90% of total PARylation activity in response to DNA damage and consequently is a major NAD^+^ consumer (117), reduced PARP1 content may result in increased intracellular NAD^+^ supply for other key metabolic processes such as glycolysis, TCA cycle activity, and SIRT deacetylase activity. These findings require further investigation.

Mitochondrial enzyme activities of CS and β-HAD were augmented following 3 wk of SIT in all nutritional groups, with no effect of nutritional condition. Previous studies demonstrate increased CS activity following exercise training, including as little as six sessions of SIT (28, 30–33). The potential efficacy of fasted exercise on mitochondrial enzyme activities such as CS, β-HAD, and other surrogate markers such as succinate dehydrogenase (SDH) have been examined, of which some studies have implemented endurance training (28, 118), and others HIIT (30, 119) as the primary exercise modality. However, these studies have all evaluated mitochondrial enzymatic activity changes compared with CHO-fed conditions, and no previous work has investigated any potential effect of protein supplementation on such markers. Our findings suggest no effect of preexercise protein supplementation on the long-term enzymatic activity changes measured here.

### Performance

Augmented aerobic and anaerobic exercise performance was observed following 3 wk of SIT, irrespective of nutrient condition. Previous studies have determined the effects of co-ingesting protein and CHO on aerobic exercise performance compared with CHO intake alone, with evidence suggesting an additive effect of protein intake on time-to-exhaustion, but not time trial performance, which is comparable with what was conducted here (120). Of studies investigating the effects of protein supplementation alone on training-induced aerobic exercise performance adaptations, available evidence indicates no additive effect compared with either noncaloric placebo (121) or CHO ingestion (122). Despite some differential metabolic adaptations, our results mirror these findings, albeit following a SIT intervention. Training-induced changes in anaerobic mean power were not significantly different between fasted, WPC, and WPH groups, although moderate-to-large between-groups effects were observed favoring the WPH group, compared with both fasted and WPC groups. However, when anaerobic power output data were stratified temporally per 5 s, differences in the pattern of adaptive response were observed which included a difference between WPH and WPC groups from 16 to 20 s favoring WPH. The WPH group showed a more consistent rate of adaptation across the 30 s Wingate test compared with other groups, reflected by the within-group significant increases in power output at each 5-s stage. In addition, the post-training improvement in fatigue index in WPH conditions suggests a greater ability to sustain high-intensity exercise performance in this group compared with WPC conditions, where a moderate between-groups effect size was observed. Few studies investigating the effects of protein supplementation on anaerobic exercise performance are available in the extant literature. One previous study demonstrated a superior training-induced increase in Wingate peak power following soy protein supplementation in judo athletes compared with habitual diets (123). These findings are indicative that protein supplementation does not compromise the training-induced adaptations in Wingate performance, and that WPH may enhance anaerobic performance, particularly during sustained periods of high-intensity exercise as reflected by the more consistent increases in anaerobic power output across the entire 30-s Wingate test and the favorable improvements in fatigue index in WPH conditions. These data although interesting, are preliminary in nature and require further investigation.

### Conclusions

This study presents novel findings related to the metabolic adaptations to acute (single bout) and chronic (3 wk) SIT performed under different nutritional conditions, including information on the serum metabolome, skeletal muscle signaling and transcriptomics, and exercise performance. Furthermore, this is the first study comparing hydrolyzed and intact protein supplementation for supporting adaptations to SIT. Supplementation of protein before an acute bout of SIT results in differential signaling, suppressing exercise-induced increases in pan-acetylation and decreased PARP1 protein content, while increasing amino acid supply. Interestingly, resting muscle acetylation status was downregulated in WPH conditions following training. Protein ingestion did not blunt the metabolites typically expected to be elevated during fasted exercise, and specific metabolites were also augmented to a greater extent in WPH compared with other nutritional conditions. Preexercise protein has no negative impact on skeletal muscle mitochondrial gene expression adaptations to SIT compared with exercising in the fasted state. In addition, WPH ingestion beneficially regulates exercise-induced *CD36* mRNA expression. Despite these divergent metabolic adaptations, we observed similar training-induced increases in mitochondrial enzymatic activity and aerobic performance across nutritional groups following 3 wk of SIT. Although there is no effect on mean power output adaptations, some components of anaerobic performance appear to be augmented in the WPH group. Potential limitations of our study include that for some measures, effect sizes suggest we may have been slightly underpowered in terms of sample size to adequately detect between-groups differences of biological significance. The parallel groups design employed in this study contributed to this overall reduction in power, though a crossover design was not possible given the constraints of the project. Moreover, though we piloted the intervention length and observed changes in performance markers, a longer duration may have better elucidated divergent metabolic and performance adaptations to SIT between nutrient conditions. The efficacy of both the fasted state, and preexercise protein feeding, on exercise-induced mitochondrial adaptations in skeletal muscle following sprint-based exercise will require further research to fully elucidate our initial findings.

## SUPPLEMENTAL DATA

10.6084/m9.figshare.15029316.v1 Supplemental File S1: https://doi.org/10.6084/m9.figshare.15029316.v1.

## GRANTS

This research was supported by a grant (Contract No. 7867835.4 from Carbery Food Ingredients Ltd. (to B. P. Carson), which supported a studentship and subsequent fellowship for T. P. Aird. A. J. Farquharson and J. E. Drew are supported by the Scottish Government’s Rural and Environment Science and Analytical Services Division.

## DISCLOSURES

No conflicts of interest, financial or otherwise, are declared by the authors.

## AUTHOR CONTRIBUTIONS

T.P.A., A.J.F., A.O., J.E.D., and B.P.C. conceived and designed research; T.P.A. and A.J.F. performed experiments; T.P.A., A.J.F., K.M.B., A.O., J.E.D., and B.P.C. analyzed data; T.P.A., A.J.F., K.M.B., A.O., J.E.D., and B.P.C. interpreted results of experiments; T.P.A. and K.M.B. prepared figures; T.P.A. and B.P.C. drafted manuscript; T.P.A., A.J.F., K.M.B., A.O., J.E.D., and B.P.C. edited and revised manuscript; T.P.A., A.J.F., K.M.B., A.O., J.E.D., and B.P.C. approved final version of manuscript.
